# Petrophysical core-based zonation of OW oilfield in the Bredasdorp Basin South Africa

**DOI:** 10.1038/s41598-021-04447-6

**Published:** 2022-01-11

**Authors:** Mimonitu Opuwari, Blessing Afolayan, Saeed Mohammed, Paschal Ogechukwu Amaechi, Youmssi Bareja, Tapas Chatterjee

**Affiliations:** Petroleum Geosciences Research Group, Earth Sciences Department, University of the Western Cape, 7535 Bellville, Republic of South Africa

**Keywords:** Stratigraphy, Geology, Geophysics, Core processes

## Abstract

This study aims to generate rock units based on core permeability and porosity of OW oilfield in the Bredasdorp Basin offshore South Africa. In this study, we identified and classified lithofacies based on sedimentology reports in conjunction with well logs. Lucia's petrophysical classification method is used to classify rocks into three classes. Results revealed three lithofacies as A (sandstone, coarse to medium-grained), B (fine to medium-grained sandstone), and C (carbonaceous claystone, finely laminated with siltstone). Lithofacies A is the best reservoir quality and corresponds to class 1, while lithofacies B and C correspond to class 2 and 3, which are good and poor reservoir quality rock, respectively. An integrated reservoir zonation for the rocks is based on four different zonation methods (Flow Zone indicator (FZI), Winland r35, Hydraulic conductivity (HC), and Stratigraphy modified Lorenz plot (SMLP)). Four flow zones Reservoir rock types (RRTs) were identified as RRT1, RRT3, RRT4, and RRT5, respectively. The RRT5 is the best reservoir quality composed of a megaporous rock unit, with an average FZI value between 5 and 10 µm, and HC from 40 to 120 mD/v^3^, ranked as very good. The most prolific flow units (RRT5 and RRT4 zones) form more than 75% of each well's flow capacities are supplied by two flow units (FU1 and FU3). The RRT1 is the most reduced rock quality composed of impervious and nanoporous rock. Quartz is the dominant framework grain, and siderite is the dominant cement that affects flow zones. This study has demonstrated a robust approach to delineate flow units in the OW oilfield. We have developed a useful regional petrophysical reservoir rock flow zonation model for clastic reservoir sediments. This study has produced, for the first time, insights into the petrophysical properties of the OW oilfield from the Bredasdorp Basin South Africa, based on integration of core and mineralogy data. A novel sandstone reservoir zonation classification criteria developed from this study can be applied to other datasets of sandstone reservoirs with confidence.

## Introduction

Petrophysical reservoir characterization on reservoir scale or classification of the pore spaces based on pore throat size distribution is a valuable tool in dividing pore types to explain uneven relationships between permeability and porosity for different rock types^1,2^. Porosity and permeability are the two properties that are important for reservoir rock typing or zonation studies, which can be measured directly from the core. Furthermore, the core analysis results in reservoirs produce valuable datasets regarded as the ground truth measurements used for the calibration and conditioning of other petrophysical measurements such as well logs and seismic^3–7^.

Many authors successfully apply core plug analysis results of porosity and permeability in carbonate and classic environments to quantify the reservoir flow and storage character and determine reservoir quality, e.g.^8–13^. An integrated petrophysical reservoir characterization method that involves core, well logs, and sedimentology helps to improve reservoir description by investigating various rock types that ultimately leads to an enhanced oil recovery^14–17^. Several studies^18–23^ have reported that reservoir quality is controlled by the pore geometry, which determines fluid movements. The changes from diagenetic processes could alter permeability, porosity, and lithology within a reservoir that would produce zones with different reservoir properties. Therefore, reservoir rock type classification or zonation and modelling is an essential method to explore in the oil field development stage to investigate factors that influence variations in reservoir properties El Sharawy and Nabawy 2016, Opuwari et al. 2020, Nabawy et al. 2018.

The oil field investigated in this study is situated in the Bredasdorp Basin, which lies offshore on the southern flank of South Africa. The Bredasdorp Basin holds a promising hydrocarbon prospect with several exploration, development, appraisal, and production wells drilled over the years. The Lower Cretaceous age sediments have been of primary exploration interest with renewed emphasis due to the recent hydrocarbon discoveries in commercial quantities in the southern Outeniqua basin offshore, South Africa.

Despite the previous studies conducted on flow zonation in the Bredasdorp Basin^24–26^ for the western, central, and northwestern parts of the Bredasdorp Basin, all the studies are targeted at the gas field in the Bredasdorp Basin. Based on our knowledge, there is no published work regarding reservoir rock typing or flow zonation of an oil field in the Bredasdorp Basin. Consequently, this work is focused on this important but neglected field. In this study, we identified and classified lithofacies based on sedimentology reports in conjunction with well logs. We used Lucia's Petrophysical classification method to produce three different classes of rock. An integrated reservoir zonation for the rocks is based on four different zonation methods (Flow Zone indicator (FZI), Winland r35, Hydraulic conductivity (HC), and Stratigraphy modified Lorenz plot (SMLP). In addition, quantitative mineralogy results from two wells are introduced to establish the effects of mineralogy on flow zones. The present study, therefore, also aims to produce, for the first time, insights into the petrophysical properties of the OW oilfield from the Bredasdorp Basin South Africa, based on integration of core and mineralogy data.

## Geological overview

The Bredasdorp sub-basin is a passive marginal sub-basin (associated with syn-rift half-graben) located beneath the Indian Ocean, covering approximately 18,000 km^2^. It is arguably one of the largest producing hydrocarbon Basins within Southern Africa^27–30^**.**

**Figure 1:**
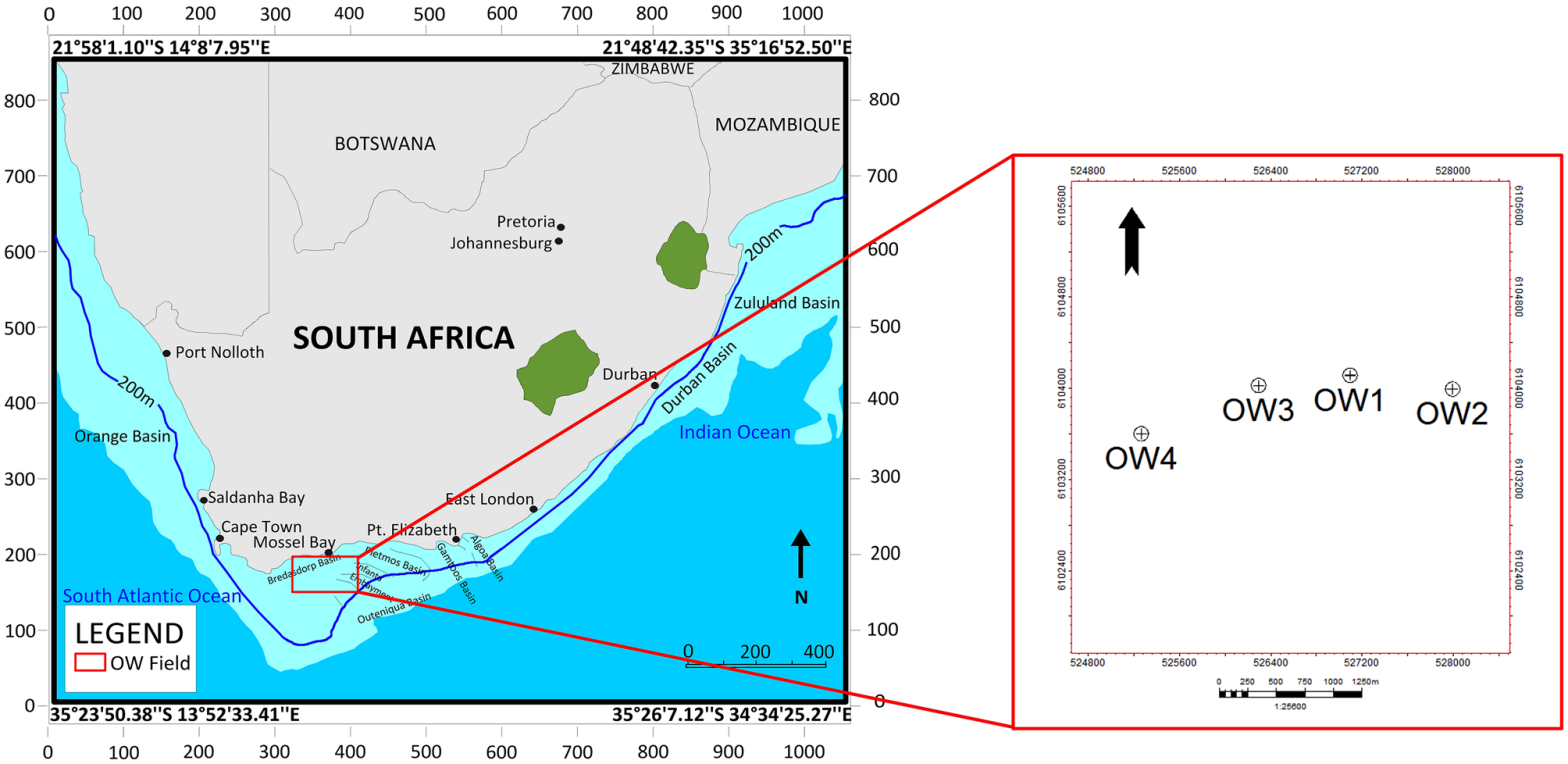
Location map generated from Petrel 2018.2 software (https://usoftly.com/products/reservoir-engineering/schlumberger-petrel-2018-2-7), showing the study area, including the well locations offshore of South Africa, modified after^32^.

The study is located in the Bredasdorp Basin, part of the larger Outeniqua sub-basin off South Africa's south coast (Fig. 1). The Bredasdorp basin has been extensively explored and is well-known for its hydrocarbon potential. The majority of South Africa's hydrocarbon discoveries and prospects have been identified in the Bredasdorp Basin. The basin is bounded on the north-eastern part by the Infanta Arch, while the Columbine-Algulhas Arch is on the western and southwestern sides^31,32^. Four wells (OW1, OW2, OW3, and OW4) are used in this study, indicated by the red text in Fig. 1.

**Figure 2:**
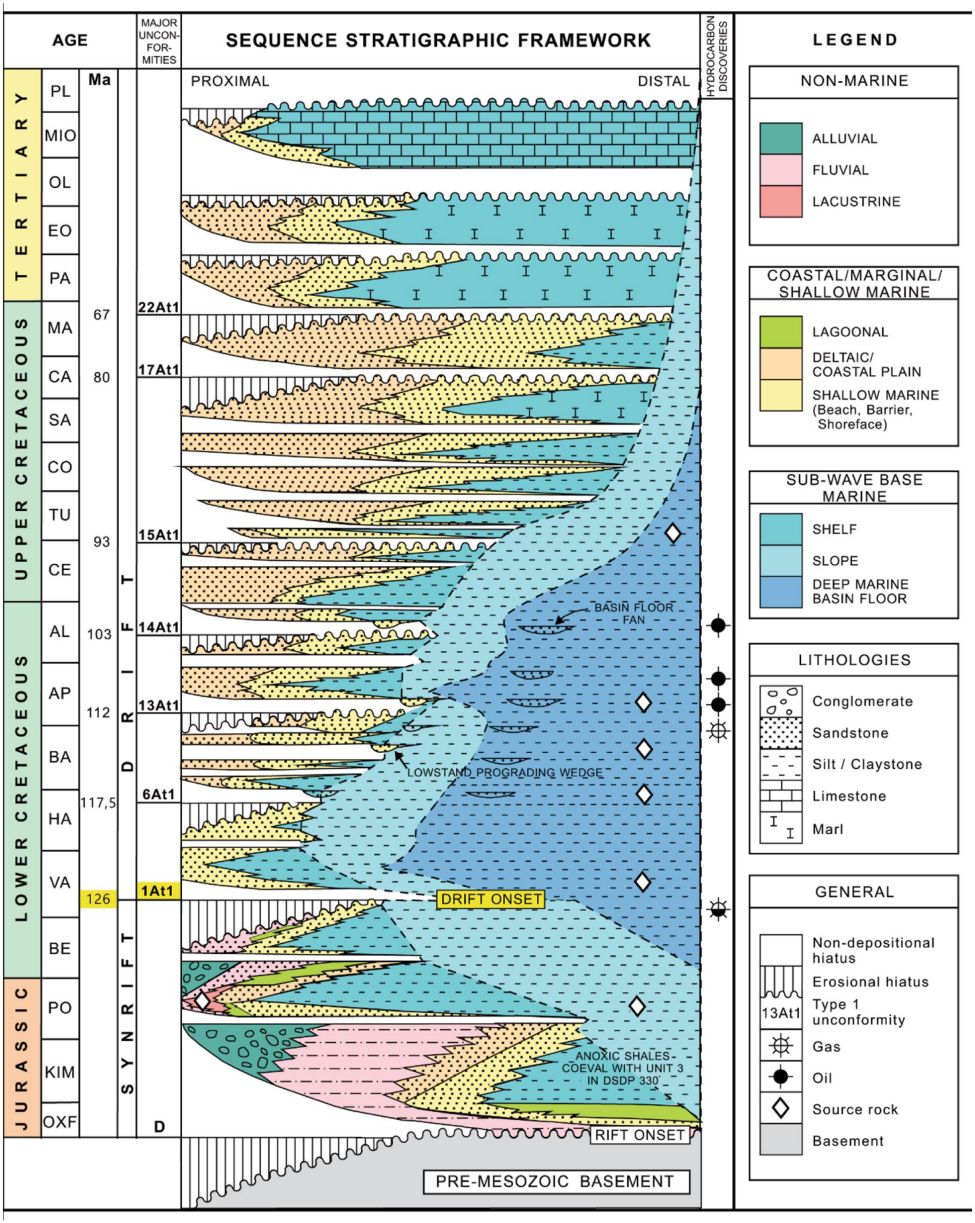
Stratigraphic nomenclature of sedimentary successions in South Africa's Offshore Basins, modified after^29,32,33^.

The stratigraphic nomenclature of sedimentary successions for South Africa's Offshore Basins is well documented. The sequences were numbered from 1 to 22 and alphabets based on major unconformities identified on seismic sections^29,33^ (Fig. 2).

According to^30^, Bredasdorp Basin sediments were derived from the erosion of the Cape Supergroup and Karoo Supergroup's sandstones and shales. The Cape supergroup sediments are made up of sequences from shallow marine, transitional, and deep-sea environments. During the Cape orogeny, the Cape supergroup was folded, generating the Cape fold belt, which extended offshore and on the coast of South Africa^34^. The Karoo supergroup was deposited atop a retro arc foreland basin when subduction and erosion began^29,35–37^. From the late Carboniferous through the early Jurassic period, the Karoo supergroup consisted of glacial, alluvial deltaic, and marine deposits^38^. There was evidence of rifting on Eastern Gondwana after the erosion of the Karoo supergroup, and syn-rift half grabens appeared^30^.

Mature shale deposited in the deep marine within the Mid-Aptian age of 13At1 in the drift section serves as the oil field's primary source rock (Fig. 2).

There are two major reservoirs in the Bredasdorp Basin. These reservoirs are shelf sandstones (syn-rift section) and deep marine turbidite sandstones (drift section)^32^ Rahiam et al. 2019. The wells in the study area encountered deep marine turbidite sandstones. The drift marine shales operate as the significant seals in the Bredasdorp basin. The seal at the Syn-rift section is non-connecting faults in tilted form and faulted blocks. Both structural and stratigraphic traps are present in the Bredasdorp Basin. Tilted fault blocks are common structural traps in the syn-rift section, while stratigraphic traps are widespread within the drift section^32,37^.

## Materials and methods

There are four wells available in the oilfield used for this study. Two cores were cut in well (OW1), with a total of 33.24 m were recovered. The core consists of deep marine sandstone with claystone at the base. Two cores were cut in well (OW2) with a 25.01 m recovery, while three cores were cut in well (OW3) with a total recovery of 28.42 m, comprise mainly sandstone with occasional thin claystone interbed. Finally, three cores were cut back to back in well (OW4) with a total recovery of 32.04 m. Routine core analyses, including helium porosity, vertical and horizontal permeability, and grain density, were conducted at ambient condition on 371 core plugs (108 for well OW1,64 for well OW2,89 for well OW3, and 110 for well OW4) that produced the porosity and permeability data used for this study.

Conventional core analysis data (Porosity and Permeability) for all wells and mineralogical data were available for two wells (OW2 and OW4). In addition, conventional well logs (gamma-ray, resistivity, density, and sonic) were available for all the wells. The evaluation process commences with the classification of lithofacies through to the delineation of reservoir zones.

In conjunction with the machine learning rock typing approach using well logs and Lucia's Petrophysical rock classification models, the Sedimentology report was adopted to group reservoir rocks into three distinct lithofacies. Four different petrophysical rock typing methods (Hydraulic conductivity (HC), Winland r35 pore throat radius Petrophysical Rock Type (PRT), Flow Zone Indicator (FZI), and Stratigraphy Modified Lorenz Plot (SMLP) applied for grouping of reservoir rocks into flow zones. Explanation of the methods is discussed in the result section.

## Results and discussion

### Lithofacies and Lucia's petrophysical classification

#### Lithofacies

The sedimentology report indicates that the wells' sandstone reservoirs s are compositionally and texturally homogeneous; therefore, lithofacies classification is possible. The sandstones are predominantly clean, very fine to coarse-grained, variably sorted, slightly lithic, and very slightly carbonaceous^32^. Porosity reduction is influenced by quartz overgrowth, with a minor amount of calcite infilling. The sandstones are occasionally silty.

The rock types classification into lithofacies was achieved by integrating well logs (gamma-ray, resistivity, and density) and sedimentology reports. The sediments of the studied wells are mainly of deep marine depositional environment. Therefore, a detailed core description of the sediments was obtained from sedimentology reports and previous work in the studied wells that provided a geological classification framework based on texture and grain size. As a result, three lithofacies were identified and subsequently grouped as lithofacies A, B, and C from core description and sedimentology reports. Lithofacies A is a clean, massive channel sandstone, coarse to medium grains; lithofacies B is fine to medium grains sandstone interbedded in places with siltstone, moderately sorted. On the other hand, lithofacies C is carbonaceous claystone, finely laminated with siltstone^32^.

**Figure 3:**
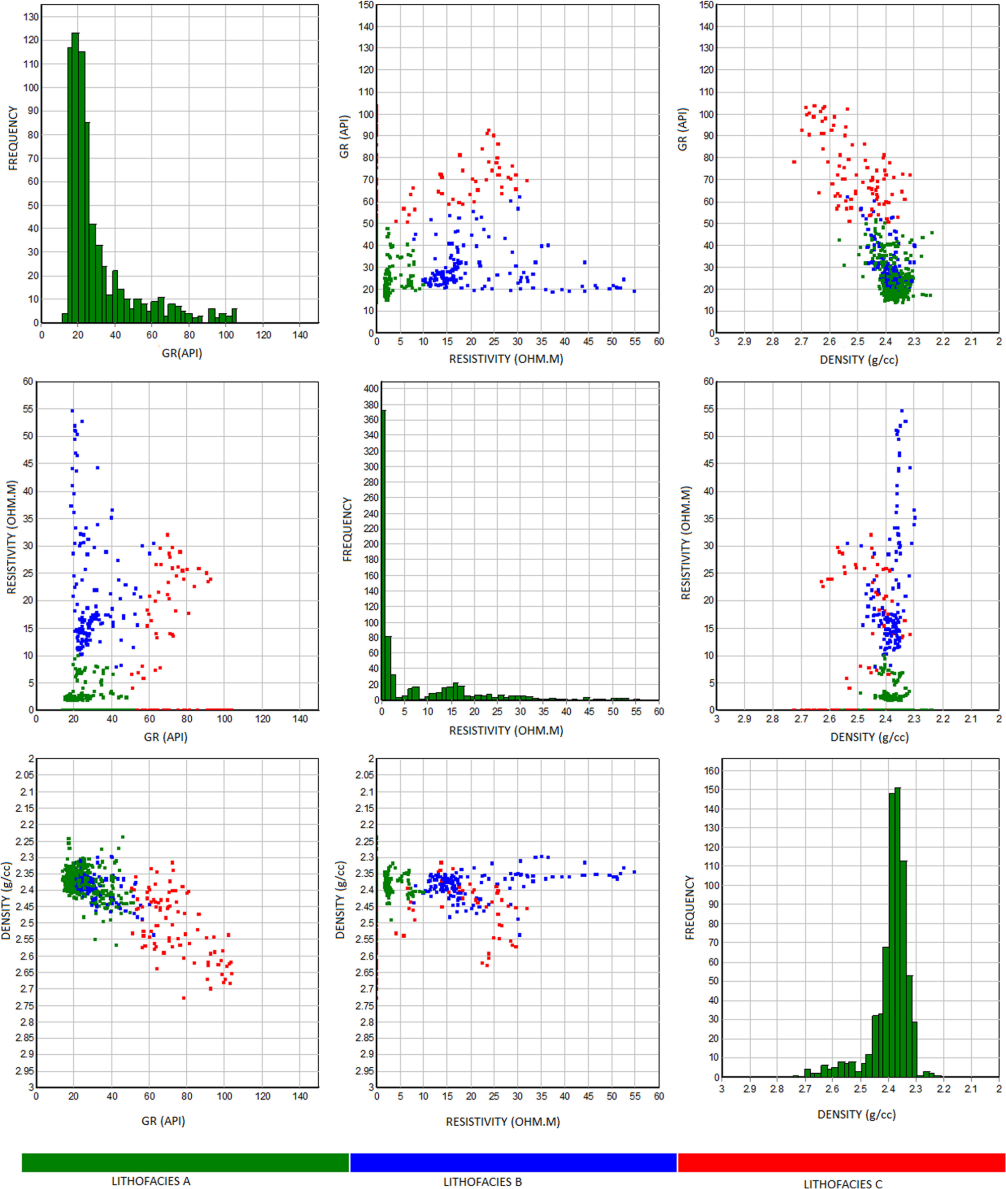
Cluster analysis showing lithofacies identified in the wells from well logs.

A machine learning rock classification algorithm using well logs (gamma-ray, density, and resistivity) from IP.4.7 rock typing module was introduced in this study to group reservoir rocks into various clusters and integrate results with core defined lithofacies. According to^39^, clustering is the method and algorithm used to cluster or group data according to measured or perceived characteristics and similarity^40^. Althogh machine learning has shown to be reliable tool for lithofacies classification, applying a single model may not produce robust result, hence the need to integrate it with discrete core data to improve the reliability and reduce uncertainities. We performed environmental corrections on the logs to improve the results of the logs to remove underlying data before maching learning operation. The results from cluster analysis for rock types are shown in Table 1 and (Fig. 3). Overlying lithofacies (facies A and B) is identified in Fig. 3 due to discriminatory criteria in the well logs used in Table 1. The number of sample points in the classification for each lithofacies is different, and such differences may affect the performance of the machine learning clustering approach Liu et al. To remove the effect and increase the reliability of the model, we integrated the results of the rock clustering method and compared to lithofacies identification by manual core examination, and sedimentology results to consolidate lithofacies rock classification as follows:

**Table 1:** Results of rock classification, clustering lithofacies into three groups.

Lithofacies	# points	GR (api)		Resistivity (Ohm m)		Density (g/cc)	
		Mean	Covariance factor	Mean	Covariance factor	Mean	Covariance factor
A	444	23.90	0.36	22.40	0.46	2.37	0.01
B	187	31.30	0.56	6.50	1.73	2.38	0.01
C	72	75.10	0.24	1.50	1.60	2.58	0.02

##### Lithofacies A

Lithofacies A is a massive sandstone, coarse to medium grains with an average gamma-ray value of 24 API, resistivity of 22 ohmm, and density of 2.37 g/cc.

##### Lithofacies B

Represents fine to medium grains sandstone interbedded in places with siltstone, moderately sorted with an average gamma-ray value of 31 API, resistivity of 7 ohmm, and density of 2.38 g/cc.

##### Lithofacies C

Carbonaceous claystone, finely laminated with siltstone with an average gamma-ray value of 75 API, resistivity of 1.5 ohmm, and density of 2.58 g/cc.

Lithofacies A is ranked as the best reservoir rock, followed by lithofacies B and C, respectively.

#### Lucia's petrophysical classification

To determine the rock type in a reservoir, it is essential to understand the primary rock properties, such as the mineralogy, texture, grain packing, and other parameters because they influence the petrophysical properties of rocks^10,12,13,17,41,42^. Lucia's Petrophysical rock classification is a technique used to relate pore size distribution to rock fabric with laboratory measurements of porosity and permeability^43^. Lucia's classification method has been successfully applied to carbonate rocks in which the pores are categorized into two broad categories: interparticle and vuggy. Rocks with interparticle pore spaces comprise three rock classes grouped as class1, 2, and 3. The grouping of the rocks is based on texture, mud-dominated or grain-dominated^44^.

**Figure 4:**
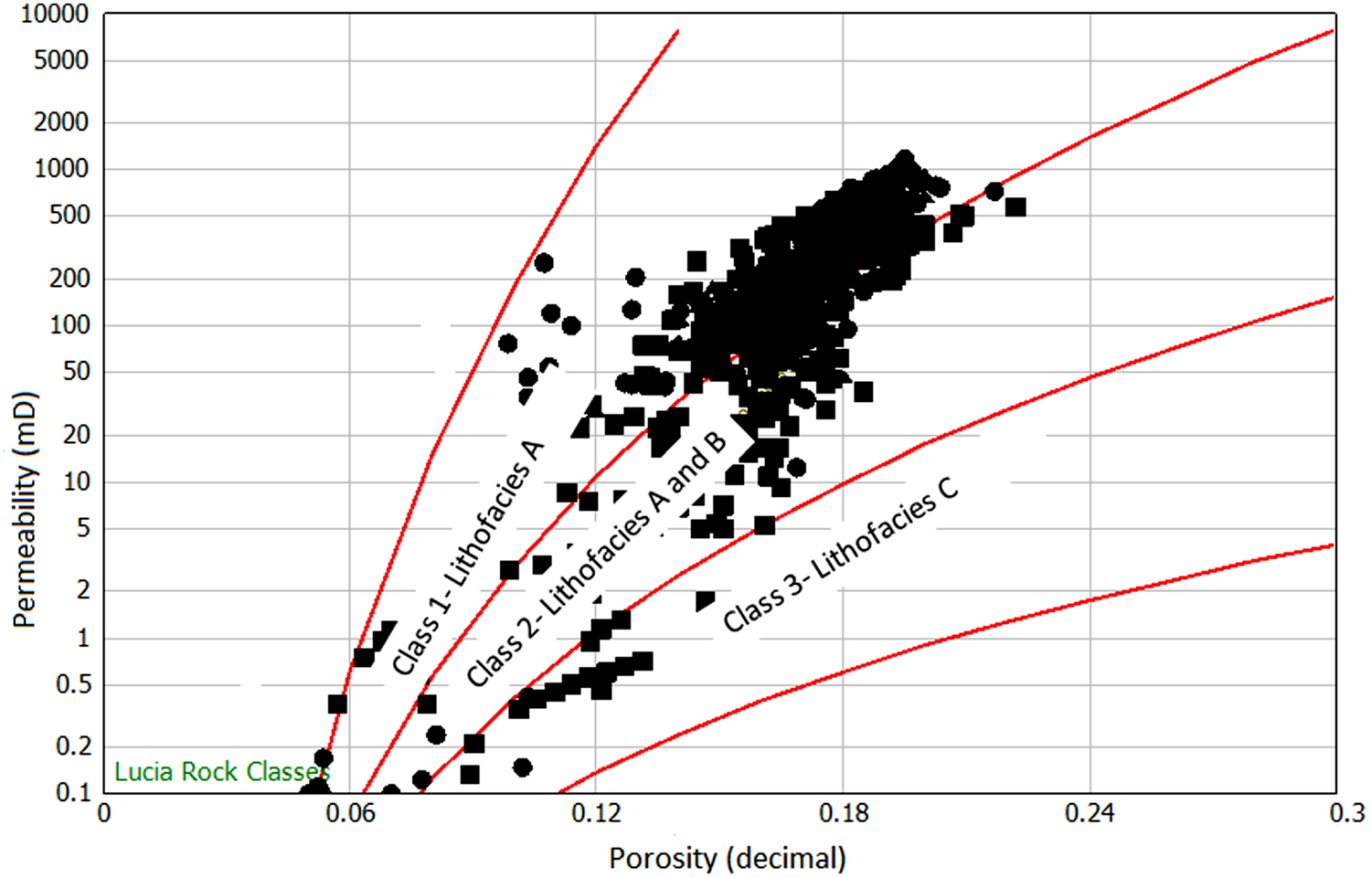
Lucia's Petrophysical rock classification showing three classes of rock.

Though Lucia's Petrophysical classification method is mainly applied to carbonate rocks, we attempted this method in this study to classify the rock types and also to establish the relationship between our data with the standard Lucia's classification by superimposing our data on the standard Lucia's classification model developed in the Interactive Petrophysics software package, IP.47. The results show that our data mostly clustered around class 1 (Lithofacies A with permeability ranging from 50 to 1000 mD, and porosity from 15 to 22%), followed by class 2 (Lithofacies B with permeability ranging from 1 to 50mD and porosity from 12 to 18%) and class 3 (Lithofacies C with a permeability of ≤ 1mD and porosity from 7 to 12%) presented in Fig. 4.

Generally, the best reservoir quality rock belongs to class1 sandstone with very good permeability and porosity. On the other hand, the least reservoir quality rock belongs to class 3, which is Lithofacies C with low permeability and fair porosity.

### Reservoir rock type classification

#### Hydraulic flow unit

The concept of the hydraulic flow unit was initially proposed from the Kozeny-Carmen equation of a capillary tube model for rock pore spaces, with a key parameter in the method as the reservoir quality index (RQI), which is the average hydraulic radius in a rock^45–48^. The input parameters for reservoir quality index (RQI) and flow Zone indicator (FZI) are core porosity (Phi) and permeability (K) data that present the relationship below:

1 $${\text{Rock}}\;{\text{quality}}\;{\text{index}}\; \left( {{\text{RQI}}} \right) = 0.0{314} \times {\text{Sqrt}}\left( {{\text{ K }}/{\text{ Phi }}} \right)$$

2 $${\text{Pore - Grain}}\;{\text{volume}}\;{\text{ratio}}\;\left( {{\text{PhiZ}}} \right) = {\text{Phi}}/\left( {{1} - {\text{Phi}}} \right)$$

3 $${\text{Flow}}\;{\text{Zone}}\;{\text{Indicator}}\;\left( {\text{ FZI}} \right) = {\text{RQI}}/{\text{PhiZ}}$$

From a log–log plot of RQI against PhiZ, we can determine points of similar FZI characteristics of value because they plot on a similar line with identical flow characteristics and identify flow zone boundaries. Therefore, we first select flow unit boundaries based on points positioned on 45° lines with similar FZI values in the analysis. Then, we create hydraulic flow units using the FZI boundaries. This method has been successfully applied and documented by researchers in carbonate and clastic reservoirs^9,24,49^.

**Figure 5:**
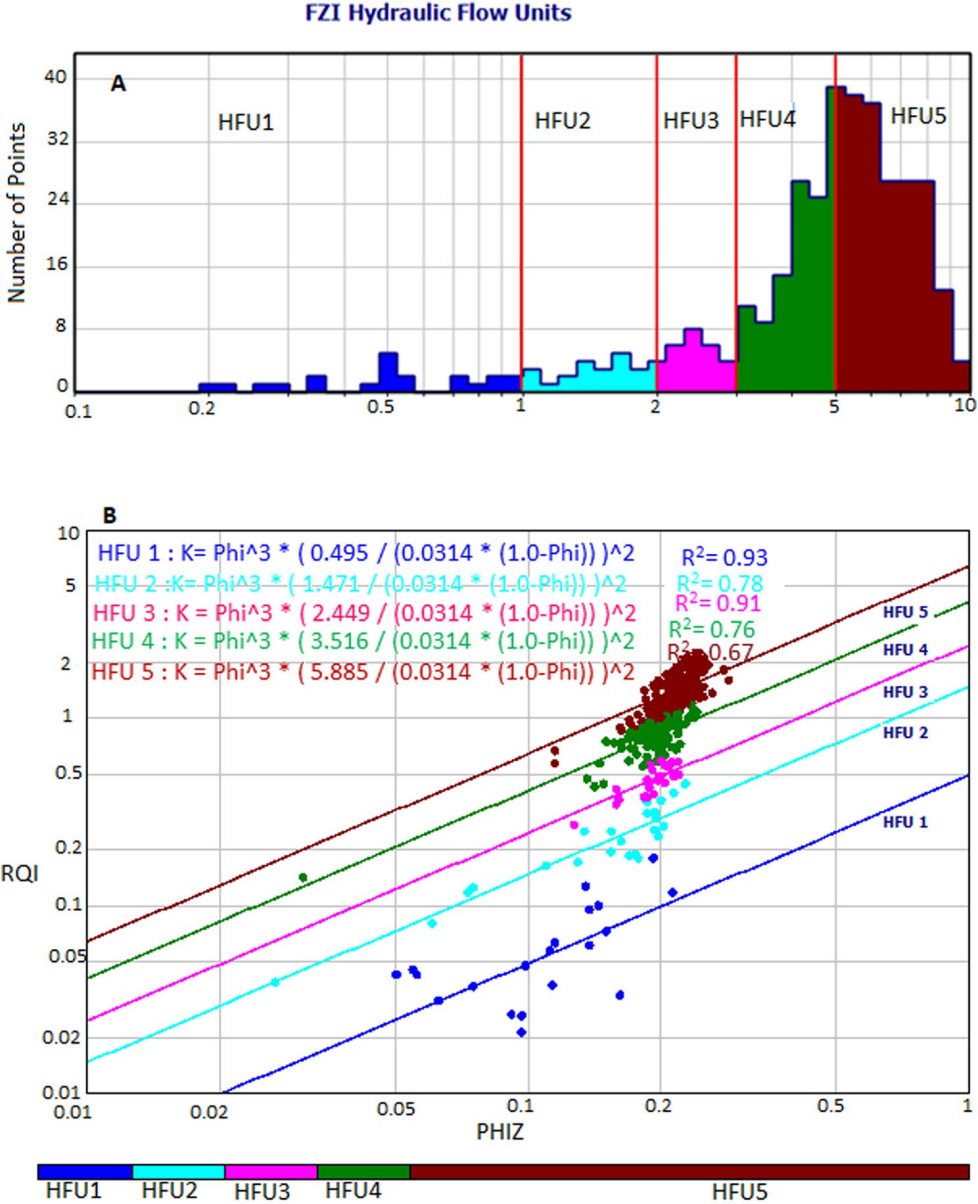

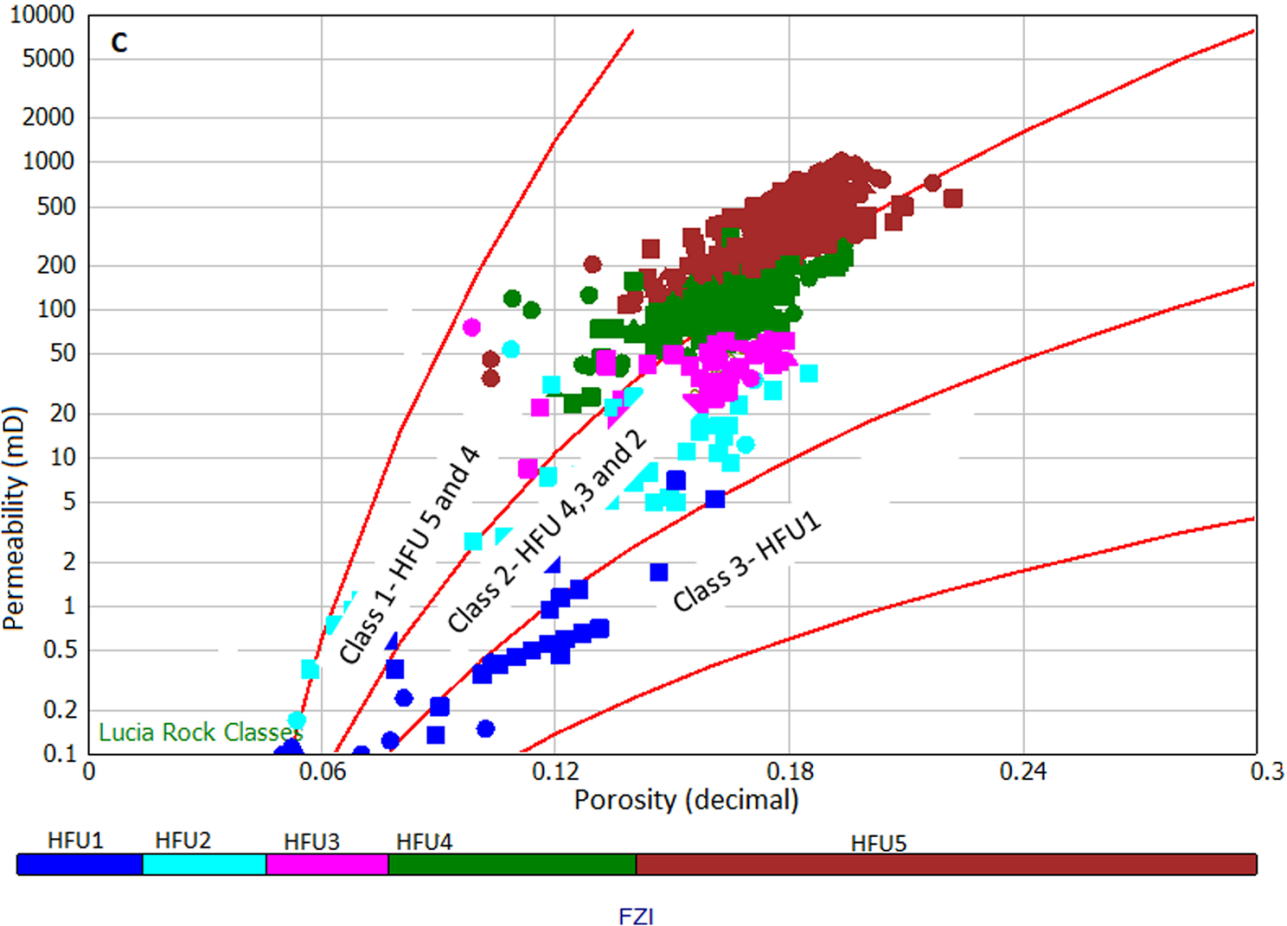
Results of flow zone indicator (**a**) Histogram of vertical boundary lines (red) showing boundaries of flow units (**b**) log–log plot of RQI versus normalized porosity (Phiz) showing five distinct flow units and respective relationships for the determination of permeability (K) from hydraulic flow unit. (**c**) Superimposition of RQI versus normalized porosity on Lucia’s plot.

Results present five flow units vertical boundary lines shown in the histogram of Fig. 5a, and the flow units are calculated using the boundaries set in the flow units statistical Table 2, which resulted in five hydraulic flow units HFU1, HFU2, HFU3, HFU4, and HFU5 shown in Fig. 5b. The boundaries using FZI indicate that HFU1 range from 0 to 1 micron, HFU 2 from 1 to 2 microns, HFU3 from 2 to 3 microns, and HFU 4 from 3 to 5 HFU 5 from 5 to 10 microns. HFU5 presents the best reservoir rock quality with RQI ranging from 0.5 to 2.1 microns, and the least reservoir rock flow unit is HFU with an RQI value ranging from 0.02 to 0.2 microns. To understand the relationship between Lucia's classes and HFUs, HFU from FZI was superimposed on the the Lucia’s plot (Fig. 5c). Results reveal that HFU5 and HFU4 belong to class 1, HFU4, HFU3, andHFU2 belong to class2, while HFU1 is represented by class 3.

**Table 2:** Calculated avarage values of petrophysical parameters used to group rock types into five Reservoir Rock Types (RRT1-5) modified after^26^.

Well	Top depth (m)	Bottom depth (m)	Thickness (m)	Porosity %	Permeability mD	Zone/RRT	r35 (μm)	HFU	Rock Type	FZI (μm)	Ranking	Hydraulic Conductivity mD/v^3^
				18–22	200–1000	RRT5	> 10	5	Megaporous	5–10	Very Good	50–120
				12–18	50–200	RRT4	4–10	4	Macroporous	3–5	Good	20–50
				10–18	10–50	RRT3	2–4	3	Mesoporous	2–3	Fair	5–20
				10–14	1–10	RRT2	1–2	2	Microporous	1–2	Poor	1–5
				< 10	< 1.0	RRT1	< 1	1	Nanoporous	< 1	Impervious	≤ 1
OW1	2624.0	2634.7	10.7	17.7	332.0	RRT5	14.0	5	Megaporous	6.4	Very Good	62.3
	2634.7	2635.8	1.1	11.3	10.0	RRT3	2.3	3	Mesoporous	2.1	Fair	6.0
	2635.8	2640.0	4.2	15.8	216.0	RRT5	13.1	5	Megaporous	6.5	Very Good	66.6
	2640.0	2648.7	8.7	15.5	104.4	RRT4	8.0	4	Macroporous	4.5	Good	30.0
	2648.7	2652.2	3.5	15.0	60.0	RRT4	6.9	4	Macroporous	3.9	Good	26.0
OW2	2579.4	2584.4	5.0	18.1	333.0	RRT5	14.7	5	Megaporous	6.4	Very Good	66.5
	2584.4	2589.0	4.6	17.8	245.0	RRT5	12.4	5	Megaporous	5.6	Very Good	51.0
	2589.0	2596.1	7.1	16.7	164.0	RRT4	9.6	4	Macroporous	4.7	Good	35.7
OW3	2616.6	2620.5	3.9	16.2	275.0	RRT5	14.6	5	Megaporous	7.5	Very Good	64.7
	2620.5	2622.7	2.2	7.0	0.4	RRT1	0.8	1	Nanoporous	0.8	Impervious	1.0
	2622.7	2636.5	13.8	17.1	304.0	RRT5	14.0	5	Megaporous	6.4	Very Good	70.0
	2636.0	2637.3	1.3	6.4	0.9	RRT1	0.9	1	Nanooporous	1.1	Impervious	1.2
	2637.3	2642.5	5.2	16.7	110.0	RRT4	8.3	4	Macroporous	4.2	Good	31.0
OW4	2609.4	2612.6	3.2	17.7	221.0	RRT5	11.5	5	Megaporous	5.3	Very Good	49.5
	2612.6	2616.4	3.8	17.7	167.0	RRT4	10.1	4	Macroporous	4.7	Good	39.5
	2616.4	2618.0	1.6	7.2	0.3	RRT1	0.4	1	Nanoporous	0.5	Impervious	0.3
	2618.0	2619.8	1.8	10.2	10.0	RRT3	3.5	3	Mesoporous	2.4	Fair	14.2
	2619.8	2623.0	3.2	4.2	0.2	RRT1	0.5	1	Nanoporous	0.9	Impervious	0.7
	2623.0	2630.0	7.0	15.8	109.0	RRT4	4.7	4	Macroporous	3.2	Good	22.0
	2630.0	2642.0	12.0	16.6	123.0	RRT4	6.9	4	Macroporous	3.6	Good	23.4

#### Winland r35 rock typing model

**Figure 6:**
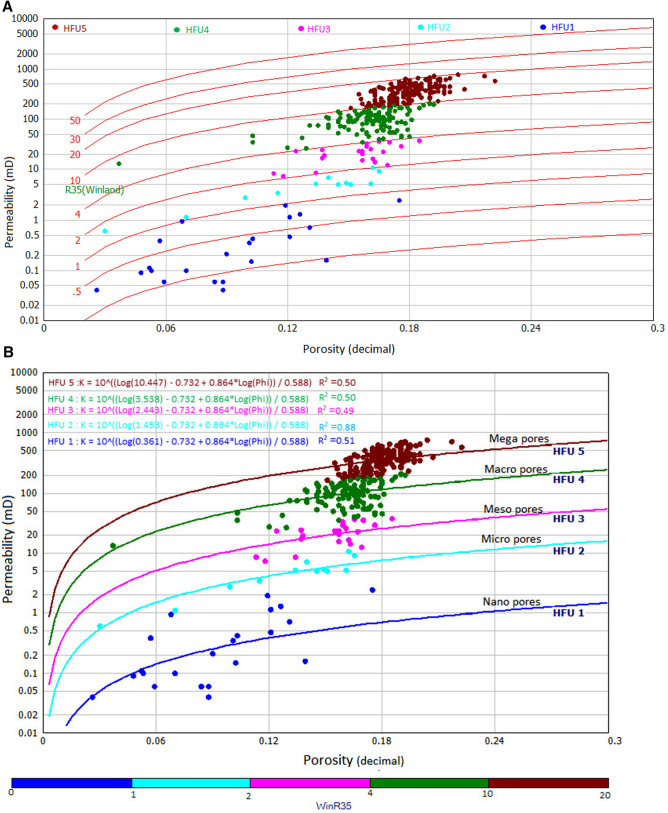

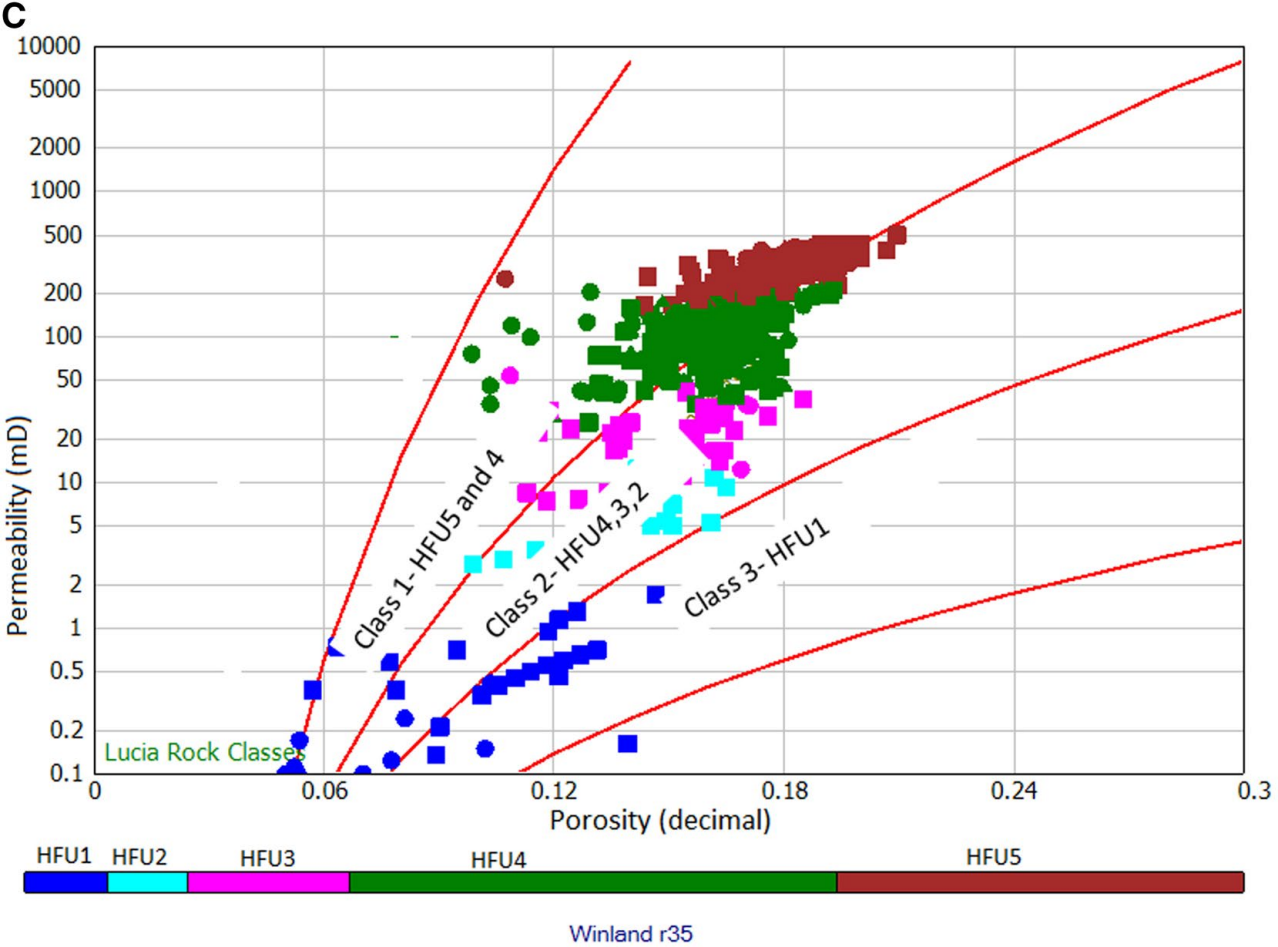
(**a**) Winland r35 pore throat radius for the identification of rock types, showing five different rock types from HFU 1 to HFU5. (**b**) Results of Winland r35 pore throat radius showing five different equations for rock types. (**c**) Superimposition of Winland r35 results on Lucia’s plot.

Winland^50^ created a method for determining pore throat radius from core data using core measurements of porosity and permeability data, published by^51^. From the Winland r35 method, the pore throat radius (r35), corresponding to the 35th percentile of mercury saturation (µm) could be calculated on different data such as well logs and core provided they have porosity and permeability data^15,51,52^. From that concept, Winland noted that large pores connected large crystals, and small pores connected small crystals. Therefore, Winland indicated that if the intercrystalline pore system is filled by intergranular and solution pore, the one that controls outflow and inflow into large pore is the smallest pore system^53^. Winland's correlation equation between pore sizes, porosity, and permeability published by^51^ is shown in Eq. (4).

4 $${\text{Winland}}\;{\text{r35}} = {\text{Log}}\left( { \, 0.{732} + 0.{\text{588 Log}}\left( {\text{K}} \right) - 0.{\text{864 Log}}\left( {{\text{Phi}}} \right)} \right)$$

where Phi is porosity (%) and K is air permeability in mD. From Eq. (4), rock typing can be done by calculating the r35 value for each sample, classifying the sample with the same r35 value, and making an iso-pore throat line in a graph. In this study, rock typing has been done for 371 plug samples from routine core analysis with Winland r35 method. Our result was superimposed on the standard Winland r35 plot shown in Fig. 6a. From the plot, we obtained distribution pore spaces, porosity, and permeability at iso-pore throat line that presents five hydraulic flow units (HFU5 as mega pores ranging between 10 and 20 µm, HFU4 as macropores between 4 and 10 µm, HFU3 as mesopore between 2 and 4 µm, HFU2 as micropores ranges between 1 and 2 µm, and HFU1 as nanopores less than 1 µm). The porosity/permeability function (K/Phi) was also introduced in Fig. 6b to understand better the influence of porosity and permeability on pore throat size distribution and connectivity. Applying the (K/Phi) plot indicates that higher reservoir quality is assigned to the mega pore rocks, whereas the lowest is assigned to the nanopore rocks (Fig. 6b). Though the plot may successfully group the rocks, the influence of diagenesis and the lithology must be considered^20,54,55^. Several empirical relationships were established from the Winland r35 pore throat method to estimate permeability from porosity. To understand the relationship between Lucia's classes and HFUs, HFU was superimposed on the Lucia’s plot (Fig. 6c). Results reveal that HFU5 and HFU4 belong to class 1, HFU4, HFU3, andHFU2 belong to class2, while HFU1 is represented by class 3.

#### Hydraulic conductivity (HFU) method

From HFU and in reference to^56^, reservoir quality index method, the hydraulic conductivity method was developed^57^ for sandstone reservoirs. This method was applied in this study because we are working on sandstone reservoirs. The technique was developed by^58^ to obtain a capillary model for a porous medium from the relationship between permeability and porosity from Eq. (6): In this study, the method developed by Scheidegger, 1957 was modified and called hydraulic reservoir unit (HRU) to replace conductivity that best fits the purpose of the study.

5 $${\text{Hydraulic}}\;{\text{Reservoir}}\;{\text{Unit}}\;\left( {{\text{HRU}}} \right) = \left\{ {{\text{Permeability}}/(1014*({\text{Porosity}})^{3} )} \right\}*0.1$$

**Figure 7:**
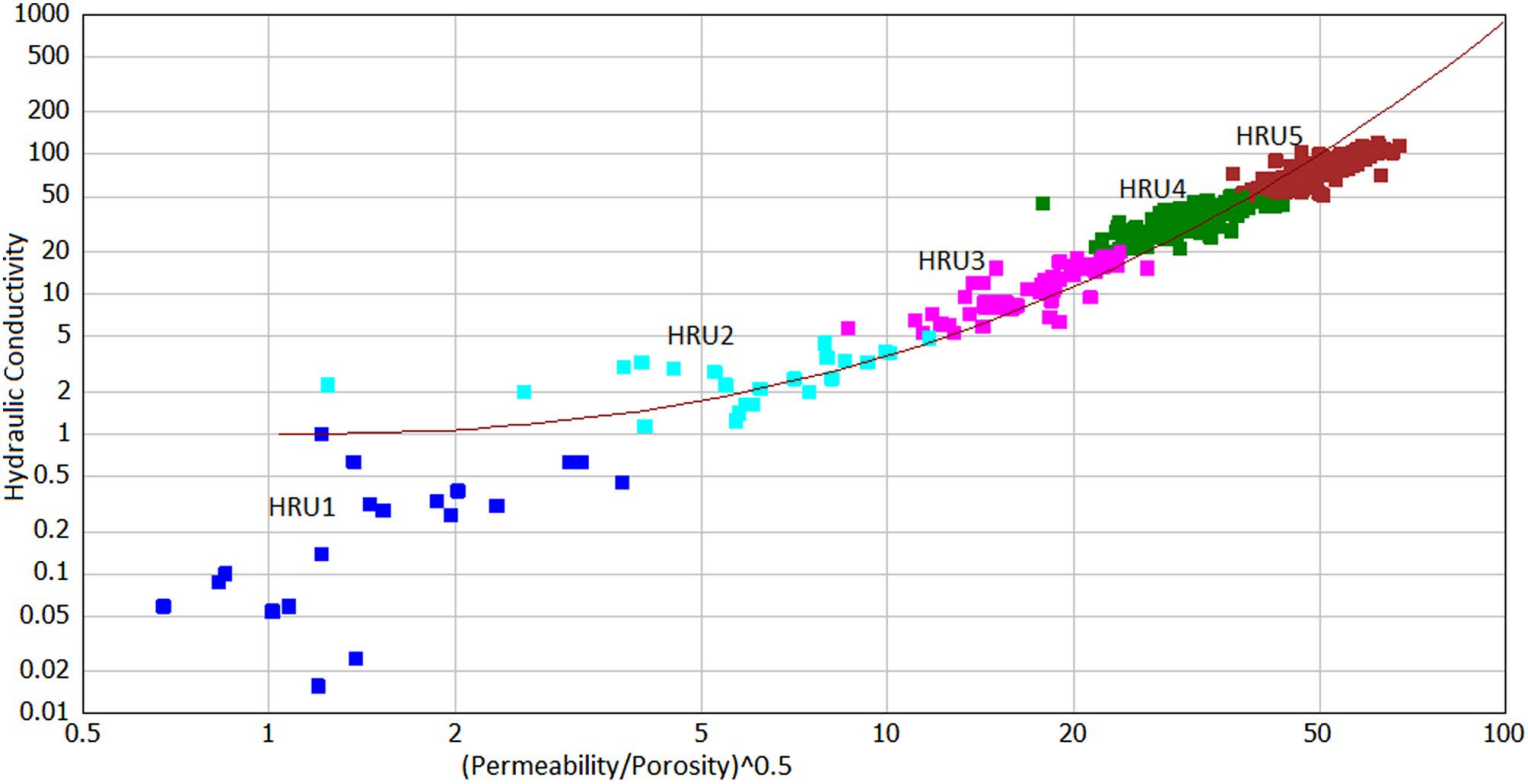
Cross plot of hydraulic conductivity against K/Phi indicating hydraulic reservoir units encountered in the wells.

In applying the hydraulic conductivity method, we first calculate HRU from each core sample using Eq. (5), then cross plot of hydraulic conductivity against (Permeability/Porosity)^0.5^ and establish five different HRU's (Fig. 7). HRU5 ranges from 50 to 120; HRU4 from 20 to 50; HFU3 from 5 to 20; HRU2 from 1 to 5; and HRU1 less than 1. The best reservoir quality is HRU5, and the least reservoir rock quality is HRU1. An empirical power relation was established to estimate hydraulic conductivity as follows:

6 $${\text{Hydraulic}}\;{\text{Conductivity}} = 0.56*{\text{Log}}(({\text{Permeability}}/{\text{Porosity}})^{0.5} )^{2.39} \;\;\;R^{2} = 0.91$$

#### SMLP method

Stratigraphic Modified Lorenz Plot (SMLP), a petrophysical-based method, was also used in this study to identify hydraulic flow units within a sequence-stratigraphic framework^59–61^. This method is applied through analysis of porosity and permeability to establish the vertical variation of flow (permeability with the thickness, kh) and storage capacity (porosity with the thickness, Ph). The SMLP utilizes the cross plot of cumulative flow and storage capacity values to determine flow units within a stratigraphic framework from base to top of reservoir^61,62^. Significant inflection points on the SMLP are interpreted to represent changes in flow and storage capacity flow unit. The interval of cumulative flow and storage capacity that slopes higher than 45° lines on the plot is used to indicate high flow and low storage capacity; interval with a slope lower than the 45° lines on the plot represents higher storage and low flow capacity, while those that plot around the 45° lines represents an interval of equal flow and storage capacity.

**Figure 8:**
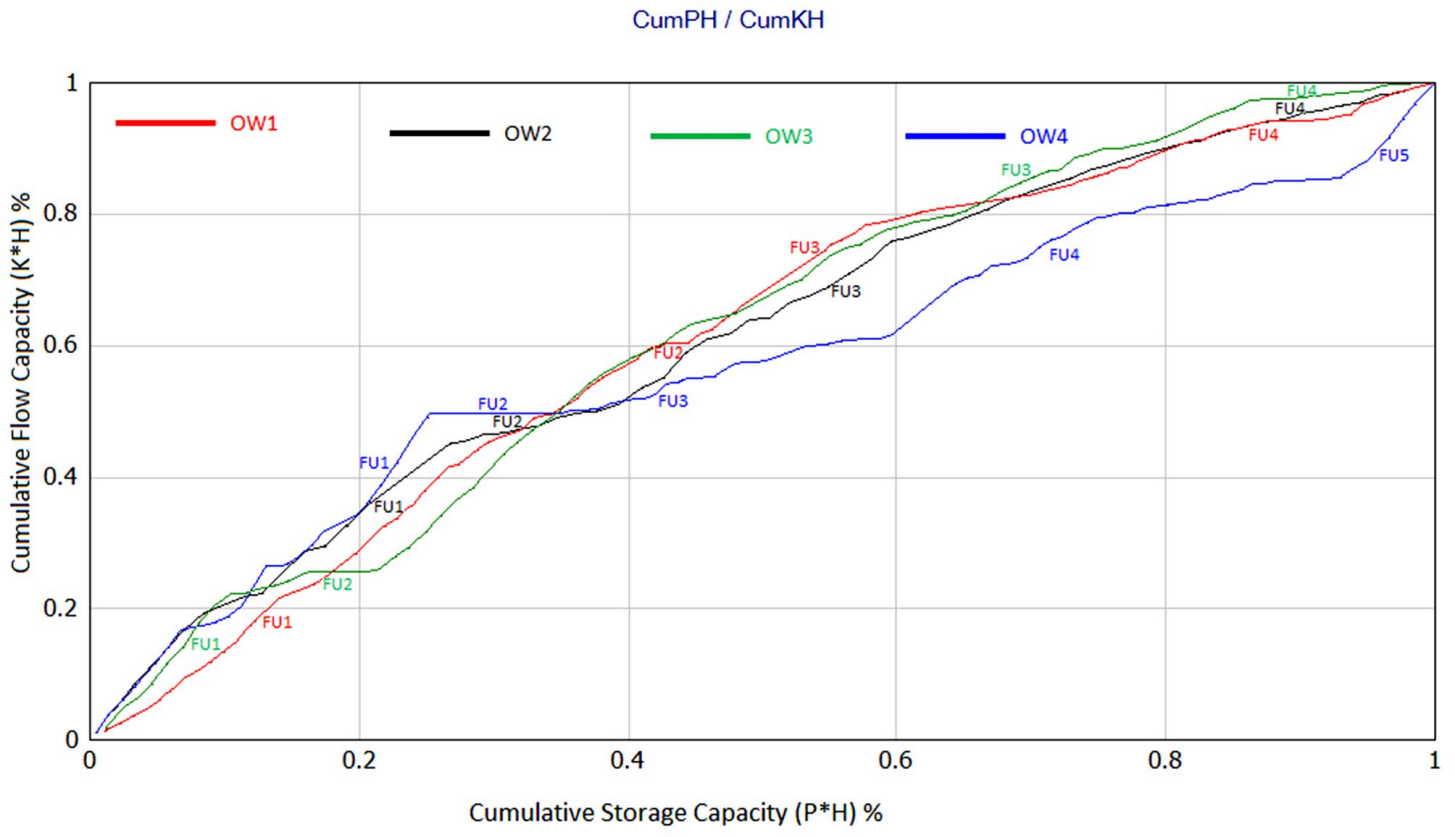
Stratigraphic modified Lorenz plots (SMPLs) for the studied wells showing different flow units (FUs).

SMLP was generated for our study using porosity and permeability values (Fig. 8). According to SMLP, four flow units (FU1 to FU4) are recognized in well OW1(red line), OW2 (black line), OW3 (green line), and five flow units (FU1 to FU5) are recognized for well OW4 (blue line) respectively. The Improved Stratigraphic Modified Lorenz plot proposed by Maglio-Johnson 2000 and El Sawy et al. 2020 was integrated with SMLP to estimate the contribution of individual flow units. As seen in Fig. 8, FU1 and FU3 contributes 91% of the flow capacity of well OW1, while FU1, FU3, and FU4 supplied 96% of flow capacity for well OW2. In well OW3, 93% of flow is supplied by FU1 and FU3, while in well OW4, FU1 and FU3 contributes to 94% of flow capacity. Generally, FU1 and FU3 presents the best reservoir quality with a high flow unit in all the wells, whereas FU2 represents the least reservoir quality and poor flow unit which are a barrier to flow.

### Reservoir Zonation model

Integrating the lithofacies, Lucia's Petrophysical rock classification model with different reservoir characterization methods developed culminated into a new applicable reservoir zonation scheme for the oil field presented in Table 2. We used an average value of the different reservoir zonation methods to produce five distinct flow zones as reservoir rock types (RRTs), as RRT1, RRT2, RRT3, RRT4, and RRT5, respectively. The RRT5 porosity and permeability values range from 18 to 22% and permeability from 200 to 1000 mD, with Winand r35 values ≥ l0 µm; FZI between 5 and 10 µm, HC between 50 and 120, which corresponds to HFU5, where the highest and the best reservoir quality is detected. Therefore, it is ranked as very good (Table 2). The RRT4 porosity and permeability values range from 12 to 18% and permeability from 50 to 200 mD, with Winand r35 values ranging from 4 to l0µm; FZI between 3 to 5 µm, HC between 20 and 50, which corresponds to HFU4, ranked as good reservoir quality. RRT3 porosity and permeability values range from 14 to 18% and permeability from 10 to 50mD, with Winand r35 values ranging from 2 to 4 µm; FZI between 2 and 3 µm, HC between 5 to 20, which corresponds to HFU3, ranked as fair reservoir quality. RRT2 is not detected in the studied samples. RRT1 is classified as an impervious reservoir rock with porosity and permeability values less than 10% and 1.0 mD, respectively. Winand r35, FZI, and HC values are ≤ 1 µm for RRT1, which corresponds to HFU1, with the least reservoir quality. We observed permeability variations at similar porosities in some flow zones, attributed to pore type control on fluid flow. It has been reported previously that sandstone samples with similar porosities can have different permeability resulting from the impact of diagenetic overprints on the pore throat radius of sandstones^63,64^. Consequently, we used quantitative point-count mineralogy results of well OW3 and OW4 (Table 3) to determine mineral types and abundances in different zones that affect reservoir quality, resulting in diagenetic effects on the pore throat radius.

**Table 3:** Result of quantitative mineralogical analysis of wells OW3 and OW4 indicating the dominant cement and clay that affects flow zones.

Well	Zone/RRT	Sample depth m	Framework Grain					Cements				Clay	
			Quartz %	Feldspar %	Lithics %	Glauconite %	Carbonaceous %	Quartz overgrowth %	Dolomite %	Siderite %	Pyrite %	Kaolinite %	Illite %
OW3	RRT5	2613.0	81.8	3.0	3.1	1.3	–	7.9	–	1.3	–	1.6	–
OW3	RRT5	2620.2	80.5	2.0	2.5	0.3	–	11.4	–	1.0	–	1.8	0.5
OW3	RRT4	2625.7	80.0	2.0	1.5	1.0	–	10.9	–	1.4	0.5	2.0	0.2
OW3	RRT4	2634.8	77.8	2.5	2.0	1.7	–	12.1	–	0.7		2.5	0.7
OW4	RRT5	2615.4	79.7	2.3	2.8	1.8	Traces	10.6	1	1	0.5	Traces	0.3
OW4	RRT4	2615.7	79.8	2.9	6.2	1.3	Traces	7.9	0.3	0.3	–	1.3	–
OW4	RRT4	2616	77.6	3	3	2	0.3	10.3	1	1	0.5	1	0.3
OW4	RRT1	2617.3	52.3	3.2	1.1	2.2	5.8	18.8	1.1	6.2	2.6	4.1	2.6
OW4	RRT1	2622.7	65.1	3.7	3.5	1	1.3	18.2	0.9	2.2	0.4	1.7	2
OW4	RRT3	2624.9	72.0	0.8	3	1	1	16.8	0.5	1.8	0.3	1.5	1.3
OW4	RRT3	2625.4	76.7	0.9	2.3	–	1.2	14.9	0.7	0.9	0.5	1.4	0.5
OW4	RRT4	2641.8	82	2.4	0.5	–	–	14.6	0.5	–	–	Traces	Traces

The results indicated quartz as the dominant framework grain ranging in abundance from 52.3% to 82 wt% (Table 3). Feldspar, lithics, and glauconite were all identified in varying proportions in the studied samples. According to^65,66^, the main factors that influence the permeability and porosity of sandstone reservoirs are the clay type and distribution, cementation, sandstone composition, hydrocarbon saturation, and compaction. Quartz overgrowth and siderite are the predominant types of cement in the samples ranging from 7.9% to 18.8% and 0.9% to 6.2 wt%, respectively. Dolomite is variable and minor pyrite is present in some samples as an alteration and replacement product. Samples of high siderite content ≥ 2% belong to the RRT1 flow zone, while samples with low siderite content ≤ 2% belong to the RRT3, RRT4, and RRT5 flow zones. The most abundant clay mineral is the kaolinite ranging from 1% to 4.1%, and illite ranging from traces to 2.6%. The amount of cement (siderite) and clay minerals (kaolinite and illite) increases in the RRT1 flow zone compared to other zones. Due to the coarse nature of kaolinite and its position on the pore spaces and not in the pore throat, kaolinite is less destructive of permeability than other clay minerals^65,67^. However, there appears to be a slight increase of illite, but the illite fraction is not sufficient to differentiate the reservoir into different RRTs.

**Figure 9:**
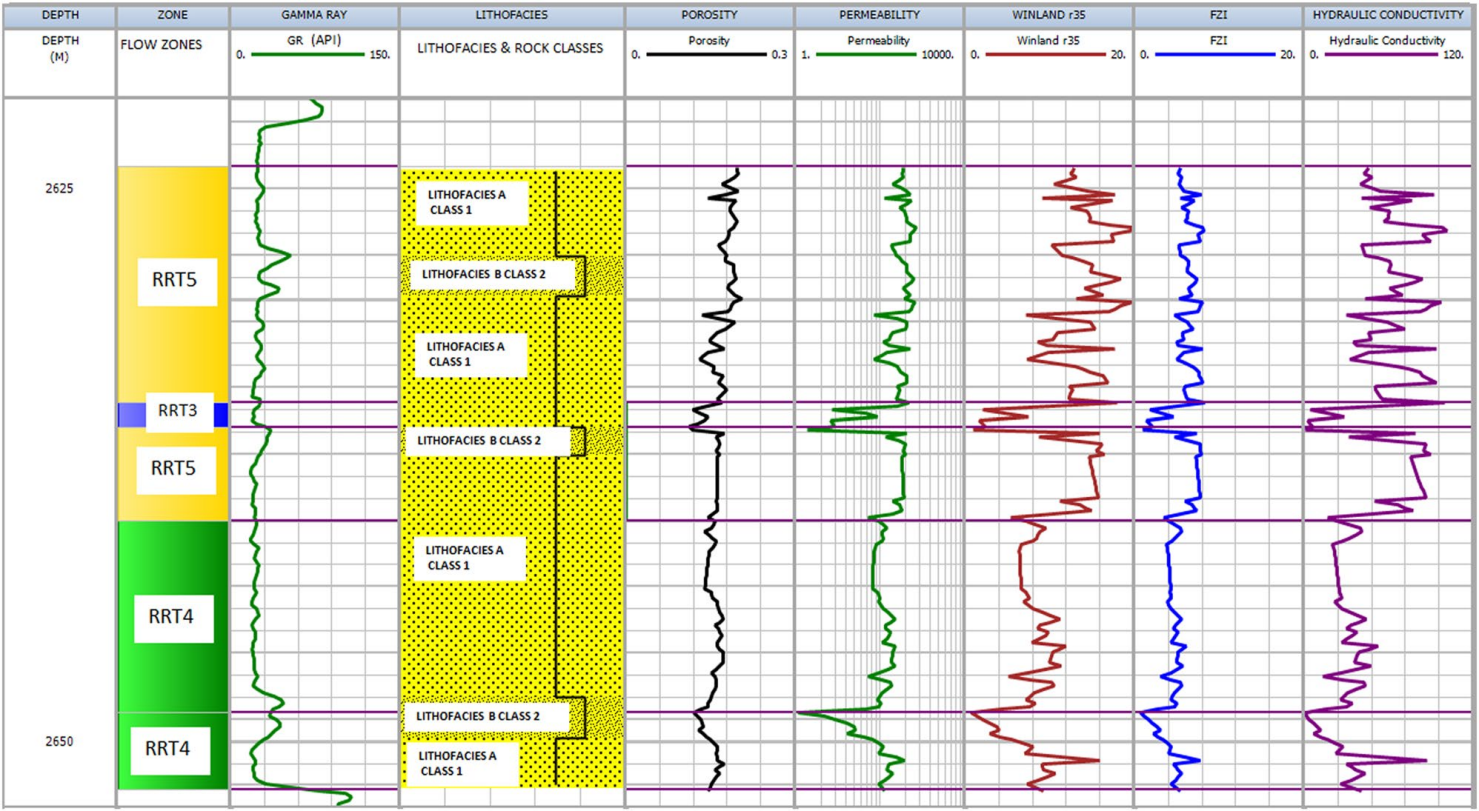
Shows the results for well OW1. Track 1 shows the measured depth. Displayed in tracks 2–9 are flow zones, Gamma ray log, lithofacies, porosity, permeability, Winand r35, Flow zone indicator, and hydraulic conductivity.

**Figure 10:**
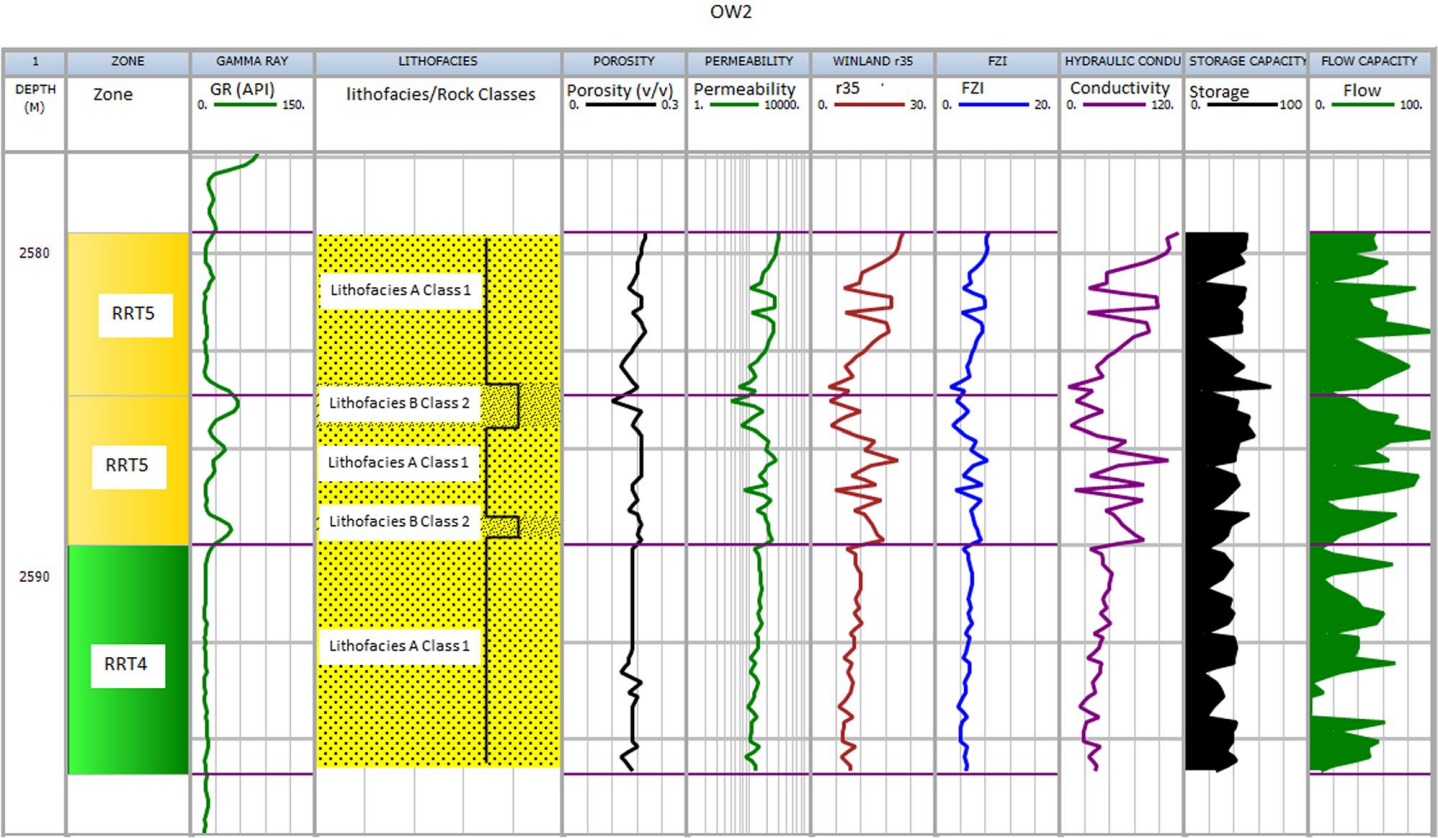
Shows the results for well OW2. Track 1 shows the measured depth. Displayed in tracks 2–9 are flow zones, Gamma ray log, lithofacies, porosity, permeability, Winand r35, Flow zone indicator, and hydraulic conductivity.

**Figure 11:**
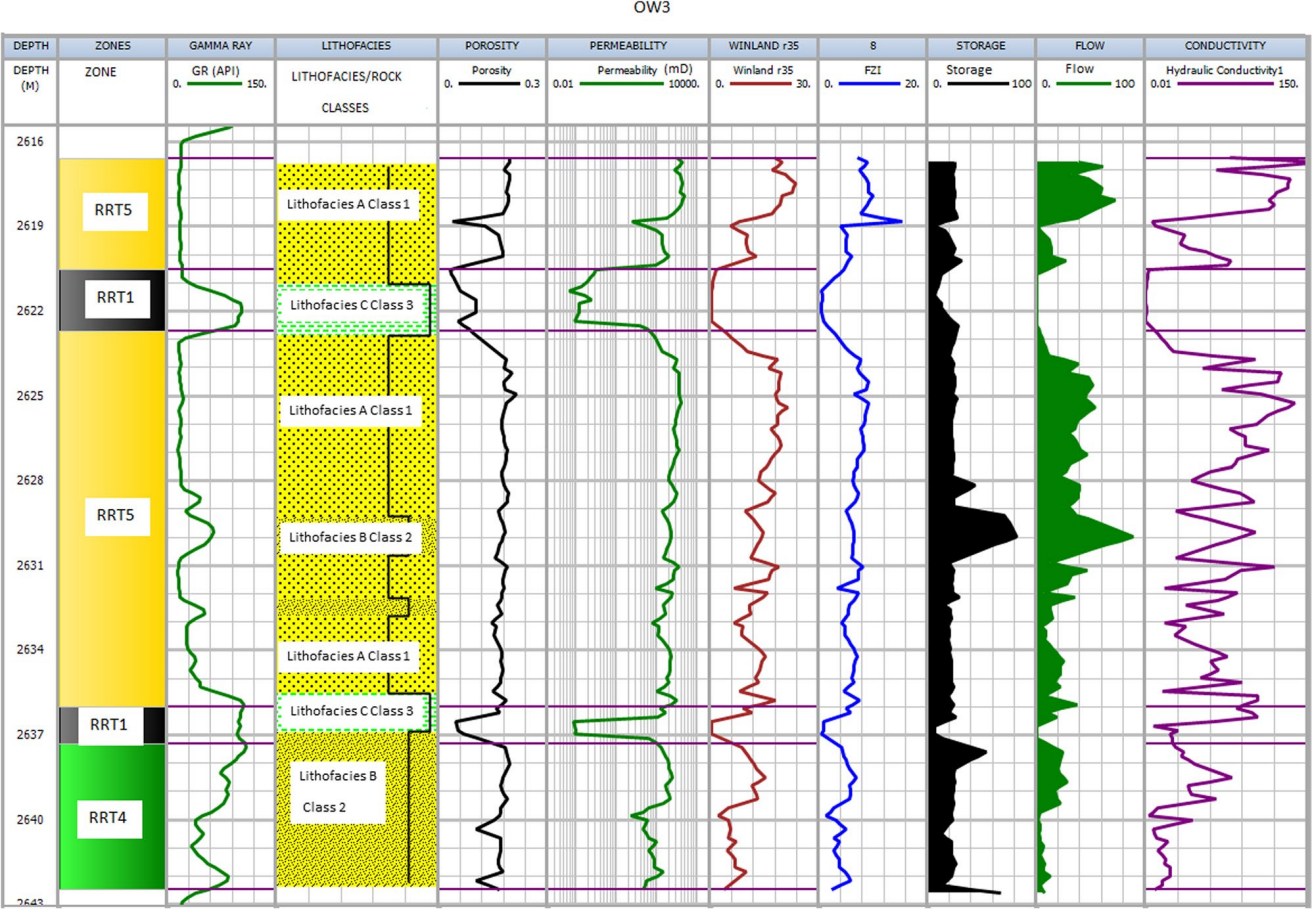
Shows the results for well OW3. Track 1 shows the measured depth. Displayed in tracks 2–9 are flow zones, Gamma ray log, lithofacies, porosity, permeability, Winand r35, Flow zone indicator, and hydraulic conductivity.

**Figure 12:**
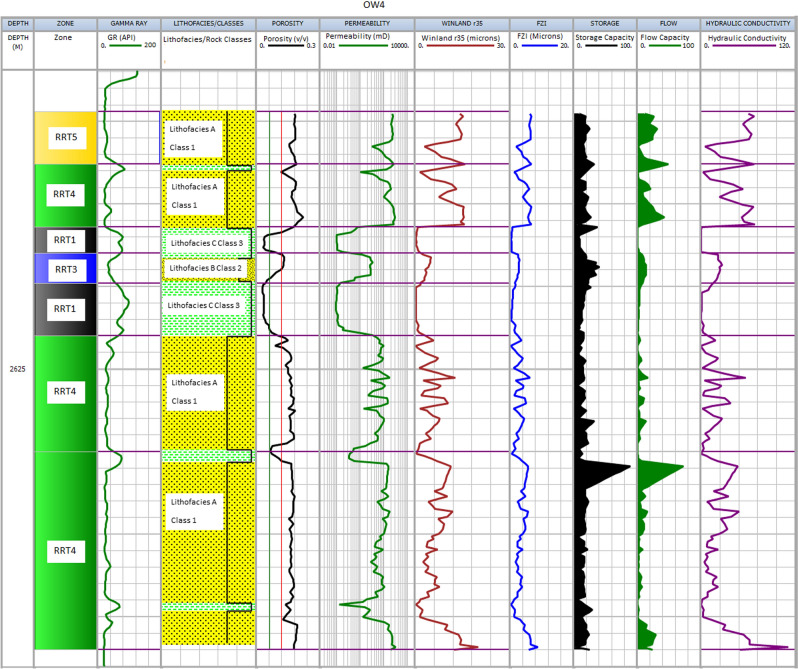
Shows the results for well OW4. Track 1 shows the measured depth. Displayed in tracks 2–9 are flow zones, Gamma ray log, lithofacies, porosity, permeability, Winand r35, Flow zone indicator, and hydraulic conductivity.

The results of integrating the four different methods (FZI, Winland r35, HC, SMLP) with lithofacies alongside each other are presented in Figs. 9, 10, 11 and 12 for each well. In addition, detailed analyses of all rock types in a zone are described in the following sections.

#### RRT5

The rocks are mainly coarse-grained sandstone (lithofacies A), and samples are, on average, moderately to poorly sorted and belong to class 1 of Lucia's Petrophysical classification. The RRT5's have very good petrophysical properties with porosity and permeability ranging from 18 to 22% and 200 mD to 1000 mD, respectively (Table 2). The pore throat size is the best, with Winland r 35 greater than 10 µm and FZI in the range between 5 and 10 µm, and HC between 50 and 120 mD/v^3^. The RRT5 is composed of HFU5 and ranked as a very good reservoir quality rock. The RRT5 appears at the upper part of the reservoirs in the studied wells (Figs. 9, 10, 11 and 12) track 2. The content of framework grain is high (80% on average), and the average content of quartz overgrowth is (10%), and siderite cement (≤ 1.3%) and illite clay mineral (≤ 0.5%) are low (Table 3). Seven RRT5's are identified in the wells. The best RRT5 is found in well OW1 (Fig. 9) with a thickness of 10 m and has strong flow capacity, FU1 (60%), and storage capacity of 42%.

#### RRT4

The rocks are generally composed of lithofacies A (coarse-grained sandstone) and B (fine to medium-grained sandstone) and belong to classes 1 and 2 of Lucia's Petrophysical classification. The RRT4's have good petrophysical properties with porosity ranging from 12 to 18% and permeability from 50 to 200mD, respectively (Table 2). The pore throat size is good with calculated Winland r 35 values range between 4 to10 µm, FZI in the range between 3 to 5 µm, and HC between 20 and 50 mD/v^3^. The RRT4 is of HFU4 and ranked as a good reservoir quality rock. Seven RRT4's are observed in the studied wells (Figs. 9, 10, 11 and 12) track 2. The best RRT4 is found in well OW2 (Fig. 10) with a thickness of 7.1 m, average porosity, and permeability of 16.7% and 16mD, respectively. The content of framework grain is high (79% on average), and the average content of quartz overgrowth is (11.2%) and siderite cement (≤ 1.4%) and illite clay minerals (≤ 0.7%) are low (Table 3). Seven RRT4's contribute more than 26% flow and 58% storage capacities, mostly from FU3.

#### RRT3

The grain size of the RRT3 is mainly composed of lithofacies B (fine to medium sandstone) of class 2 of Lucia's classification. Fair petrophysical properties are observed, with porosity ranging from 10 to 18% and permeability from 20 to 50 mD, respectively. The calculated Winland r35 pore throat size values range between 2 and 4 µm, FZI between 2 and 3 µm, and HC between 5 and 20 mD/v^3^. The RRT3 is of HFU3 and ranked as a fair reservoir quality rock. Two RRT's are observed in well OWI (Fig. 9) and OW4 (Fig. 12) track 2. The RRT3 of well OW1 is bounded vertically at the top and bottom by RRT5. The RRT3 is interpreted to provide a lithofacies A and lithofacies B contact that allows fluid flow between the high flow zones^24,68^, implying that the RRT5s are in communication. The RRT3 is identified to have the same average permeability of 10mD but differ in their average porosity values of 11.3% for well OW1 and 10.2% for well OW4, respectively. Comparison of calculated petrophysical properties between the two wells showed that OW4 has better pore throat size values (r35 of 3.5 µm, an average FZI of 2.4 µm, and HC of 14 mD/v^3^) than that of well OW1 (r35 of 2.3 µm, an average FZI of 2.1 µm, and HC of 6.0 mD/v^3^). The RRT3 is found in well OW2 (Fig. 10) with a thickness of 7.1 m, average porosity, and permeability of 16.7% and 16mD, respectively. According to^1,69^, pore throat radius is related to grain diameter, which explains why rocks of the same average permeability values may have different pore throat radius due to differences in pore volume. An average quartz framework grain of 74% and the average content of quartz overgrowth is (15.8%), and siderite cement (1.35%) and illite clay mineral (0.9%) are presented for RRT3 (Table 3). RRT3 contributes about 15% flow and 20% storage capacities, FU3 (well OW4 in Fig. 12).

#### RRT1

The essential characteristic of RRT1 is that it is predominantly composed of lithofacies C ( claystone, finely laminated with siltstone) and corresponds to class 3 of Lucia's classification. The petrophysical properties (porosity, permeability, Winland r35, FZI, and HC) of the RRT1 are ≤ 1, as shown in Table 2. An average of 63% quartz framework grain with a slight increase (1.3 to 5.8%) of carbonaceous material is indicated in this rock type. The highest amount of cement (quartz overgrowth, 18.8%, siderite, 6.2%, pyrite, 2.6%) and clay minerals (Kaolinite, 4.1% and illite, 2.6%) are indicated in the RRT1 samples (Table 3). The high amounts of cement and clay minerals ultimately fill the pores, block the pores, and constrict the sandstone's pore-throat, making the throat small, leading to poor reservoir properties observed in RRT1^70^. According to^70^, lower permeability values, as in our case (≤ 1mD), indicate that tiny pore throats play a dominant role in tight reservoirs. The pore throat is the primary physical property controlling the reservoir properties. Hence the lower the permeability, the smaller the pore throat radius.

RRT1 has the most reduced reservoir property, and it is ranked as an impervious rock that acts as a barrier to flow, found in well OW3 (Fig. 11) and OW4 (Fig. 12), respectively. As a result, the RRT1 has zero contribution to flow in both wells (OW3 and OW4) but has about 7% and 16% contributions to storage capacities (FU2) presented in Fig. 8 for both wells.

## Conclusions

An integrated study has been used to delineate the sandstone reservoirs of OW oilfield in the Bredasdorp Basin into different potential flow zones, namely: RRT1, RRT3, RRT4, and RRT5 flow zones. The petrophysical and mineralogical approach helped us understand the reservoir quality and assign flow zones to lithofacies.

The highest reservoir quality was assigned to RRT5 and lithofacies A (coarse and very coarse sandstone of Lucia's rock class I, which contains mostly megapores and is ranked as a very good reservoir rock. It is characterized by higher values for all petrophysical parameters (porosity generally between 18 and 22%, permeability between 200 and 1000mD, r35 ≥ 10 μm, FZI = 5–10 μm and HC = 50–120 mD/v^3^). The content of framework grain is high (80% on average), and the average content of quartz overgrowth is (10%) and siderite cement (≤ 1.3%), and illite (≤ 0.5%). The RRT5 is composed of HFU5, has a strong flow capacity, FU1 (60%), and storage capacity of 42%. The second best reservoir quality was the RRT4, composed of lithofacies A (coarse-grained sandstone) and B (fine to medium-grained sandstone), belonging to classes 1 and 2 of Lucia's Petrophysical classification. The RRT4's has good petrophysical properties with porosity ranging from 12 to 18% and permeability from 50 to 200mD. The pore throat size is good with calculated Winland r 35 values range between 4 and 10 µm, FZI in the range between 3 and 5 µm, and HC between 20 and 50 mD/v^3^. The RRT3 is mainly composed of lithofacies B (fine to medium sandstone) of class 2 of Lucia's classification with fair petrophysical properties. The RRT3 provides contact that allows fluid flow between RRT5s and contributes about 15% flow and 20% storage capacities, FU3. RRT1 has the least reservoir quality, and it is predominantly composed of lithofacies C (claystone, finely laminated with siltstone) and corresponds to class 3 of Lucia's classification. The highest amount of cement (quartz overgrowth, 18.8%, siderite, 6.2%, pyrite, 2.6%) and clay minerals (Kaolinite, 4.1% and illite, 2.6%) are indicated in the RRT1 samples. The high concentration of the cement and clay minerals ultimately fills the pores; it blocks the sandstone's pore-throat, making the throat small, leading to poor reservoir properties observed in RRT1 intervals.

It can be concluded that petrophysical characterization of the sandstone reservoirs of the OW oilfield has been achieved with good agreement to classify the sandstone and flow zones. This may indicate that the classification criteria can be confidently applied to other sandstone reservoirs in the basin. The present study, therefore, has produced for the first time, insights into the petrophysical properties of the OW oilfield from the Bredasdorp Basin South Africa, based on integration of core and mineralogy data.
